# Effect of Different Airborne‐Particle Abrasion Strategies on Dentin Bond Strength: An In Vitro Study

**DOI:** 10.1111/jerd.70053

**Published:** 2025-11-26

**Authors:** Rui I. Falacho, Gabriela Almeida, Romina Ñaupari‐Villasante, Joana A. Marques, Francisco Caramelo, Markus B. Blatz, Alessandro Loguercio, João Carlos Ramos

**Affiliations:** ^1^ Department of Preventive and Restorative Sciences University of Pennsylvania School of Dental Medicine Philadelphia Pennsylvania USA; ^2^ ADEMA University School University of the Balearic Islands (UIB) Palma Balearic Islands Spain; ^3^ Center for Innovation and Research in Oral Sciences (CIROS), Faculty of Medicine University of Coimbra Coimbra Portugal; ^4^ Institute of Implantology and Prosthodontics, Faculty of Medicine University of Coimbra Coimbra Portugal; ^5^ Department of Restorative Dentistry, School of Dentistry State University of Ponta Grossa Ponta Grossa Brazil; ^6^ Institute of Paediatric and Preventive Dentistry, Faculty of Medicine University of Coimbra Coimbra Portugal; ^7^ Center for Innovative Biomedicine and Biotechnology (CIBB) University of Coimbra Coimbra Portugal; ^8^ Laboratory of Biostatistics and Medical Informatics, Faculty of Medicine University of Coimbra Coimbra Portugal; ^9^ Institute of Operative Dentistry, Faculty of Medicine University of Coimbra Coimbra Portugal

**Keywords:** airborne‐particle abrasion, aluminum oxide, bioactive glass, dentin adhesion, microtensile bond strength

## Abstract

**Objective:**

To assess the influence of dentin airborne‐particle abrasion techniques on the microtensile bond strength, using two adhesive strategies (self‐etch and etch&rinse).

**Materials and Methods:**

Twenty molars were assigned to two groups (*n* = 10) according to the bonding system (Clearfil SE Bond or Optibond FL), divided into four parts and submitted to different surface treatments: no pretreatment (control), airborne‐particle abrasion with aluminum oxide without irrigation (Al_2_O_3_), airborne‐particle abrasion with aluminum oxide with irrigation (Al_2_O_3_/Aquasol), and airborne‐particle abrasion with bioactive glass 45S5 (BG45S5). Specimens underwent microtensile bond strength (μTBS) testing and scanning electron microscopy (SEM) analysis. Statistical analysis employed a linear mixed regression model, with a 5% significance level.

**Results:**

Statistically significant differences were found for both adhesive strategy (*p* < 0.001) and pretreatment (*p* < 0.001). Al_2_O_3_/Aquasol and Al_2_O_3_ exhibit statistically significant differences compared to the control (*p* < 0.001 and *p* = 0.006, respectively), while BG45S5 did not differ significantly (*p* = 0.080). SEM images reveal a smear layer with distinct density across groups, and diminished compactation whenever irrigation was used.

**Conclusions:**

Aluminum oxide with simultaneous irrigation resulted in the highest bond strength values, while without irrigation bond strength outcomes depend on the adhesive strategy. Bioactive glass did not interfere with bonding performance.

**Clinical Significance:**

Aluminum oxide airborne‐particle abrasion with concomitant irrigation presents the preferable surface pretreatment strategy when bonding to sound dentin.

## 1 Introduction

Bonding to dental tissues is possible due to an exchange process in which resin monomers replace hard dental tissue minerals. Upon polymerization, these monomers become interlocked in the created porosities, a process known as hybridization [1, 2, 3, 4, 5, 6]. The formation of the hybrid layer, through the diffusion of adhesive within the collagen network, is the major factor responsible for dentin bond strength [7, 8, 9].

When compared to enamel, the adhesion process to dentin is somewhat more complex and unpredictable [2, 3, 10]. This can be explained by the heterogeneous nature of this substrate, with a significant amount of water and collagen, apart from the hydroxyapatite [2, 11]. Additionally, an intimate connection with the pulp tissue translates into constant surface moisture and intrinsic hydrophilicity [2, 12]. Furthermore, dentin preparation leads to the formation of a smear layer containing dental tissue debris and residual bacteria [2, 5, 13]. This layer's size, composition and structure can vary, as well as the level of attachment to the underlying dentinal substrate [6, 14]. For the bonding process to occur successfully, the adhesive strategy must eliminate or dissolve and incorporate the smear layer within the hybrid layer [9, 11, 14].

Nowadays, there are two different adhesive strategies available: etch&rinse and self‐etch. Etch&rinse systems require an additional step of acid etching that, despite being a common clinical procedure, provides inherent features to the dentinal substrate that subsequently influence the bonding process [15]. By applying phosphoric acid to dentin, both extrafibrillar and intrafibrillar inorganic content is dissolved, the smear layer is eliminated and collagen fibrils become exposed [2, 16]. An alternative approach is self‐etch systems, constituted by nonrinse acidic monomers that condition and prime dentin simultaneously, demineralizing the underlying dentin, dissolving the smear layer and incorporating it into the hybrid layer [1, 2, 6, 14, 17]. Self‐etch systems allow discrepancies between the depth of demineralization and resin monomer infiltration to be avoided, preventing incomplete adhesive penetration and increased exposed collagen degradation over time [2, 18, 19].

Over the last few years, there has been a burgeoning inclination toward studying different methods for preparing dental tissues to enhance adhesive performance. These techniques, whether mechanical or chemical, encompass airborne‐particle abrasion procedures [8, 12, 18]. Airborne‐particle abrasion consists of a sharply focused stream of abrasive particles propelled by high‐velocity air pressure [7, 18, 20]. The collision between the particles and substrate causes small portions of dental tissue to be removed due to kinetic energy release [7, 18, 21, 22, 23], increasing wettability and available adhesion area [3, 7, 8, 9, 10, 18, 20, 21]. This procedure can be used for different purposes, such as carious lesions or stain removal, cavity preparation, composite repairs, and surface roughening or polishing [18, 21, 24]. The results obtained through airborne‐particle abrasion are directly correlated with particle composition, size, pressure, time of exposure, angle of incidence, tip design, and the distance of this tip to the substrate [18, 21, 25, 26, 27, 28].

According to specific clinical scenarios, different particles can be used, such as aluminum oxide (Al_2_O_3_), sodium bicarbonate, glycine, and bioactive glass [21]. Al_2_O_3_ particles are the most used nowadays due to aluminum insolubility and high biocompatibility [8]. Furthermore, alumina is chemically inert [29]. Additionally, bioactive glasses have been developed with calcium and phosphate that, through ion exchanges, suffer partial dissolution and hydroxycarbonate apatite formation when in contact with an aqueous environment [22, 29, 30]. The most used bioactive glass is Bioglass 45S5 (SylcTM, OSspray Ltd., London, UK), a bioactive calcium/sodium phosphate‐phyllosilicate, associated with the formation of a “bioactive smear layer” with reparative capacity [31, 32, 33].

The amalgamation of abrasive particles transported in a fluidic medium is gaining momentum as a favored method for optimizing dispersion control [22, 27, 34]. AquaCare (AquaCare, Velopex International, London, UK) is a standalone airborne‐particle abrasion unit, which uses an ethanol‐based fluid and several powders, including 29 μm Al_2_O_3_ and a bioactive glass powder (Sylc) [34]. Literature remains inconclusive concerning the ramifications of airborne‐particle abrasion on the dentinal substrate, thus requiring a thorough clarification of the exact consequences of this technique prior to its widespread application in clinical settings [21].

Thus, this in vitro study aims to assess the influence of three dentin airborne‐particle abrasion techniques (Al_2_O_3_, Al_2_O_3_ with irrigation and bioactive glass) on the microtensile bond strength of two different adhesive strategies (self‐etch and etch&rinse).

The tested research hypotheses were as follows:

- Different airborne‐particle abrasion techniques yield significantly different dentin bond strength values.
- Different airborne‐particle abrasion techniques result in distinct findings under scanning electron microscopy.

## 2 Materials and Methods

### 2.1 Specimen Preparation

The study complies with the ethical requirements established by the Ethics Committee of the Faculty of Medicine of the University of Coimbra (deliberation of March 11, 2025). Twenty human third molars, clinically and radiographically free of caries, cracks, restorations, root‐canal treatments or other abnormalities, extracted for orthodontic reasons from 16‐ to 40‐year‐old patients, were collected. The teeth were cleaned with periodontal scalers, polished with pumice and water to remove any residual soft tissue and calculus and immediately immersed in 1% chloramine‐T at 4°C for a maximum of 1 week. Subsequently, specimens were stored in distilled water at 4°C for a maximum of 1 month before initiating the experimental procedures (ISO/TS 11405:2015) [35].

The apical portion of the roots was removed with a diamond bur mounted in a high‐speed air turbine handpiece, with simultaneous water refrigeration, prior to pulp tissue removal with an excavator. Additionally, 5.25% sodium hypochlorite was used for irrigation, to ensure total pulp tissue and debris removal. Pulp chambers were then filled using a universal bonding system (Prime & Bond Active, Dentsply Sirona, Konstanz, Germany) and a flowable composite resin (SDR flowable composite, Dentsply Sirona, Konstanz, Germany).

**FIGURE 1:**
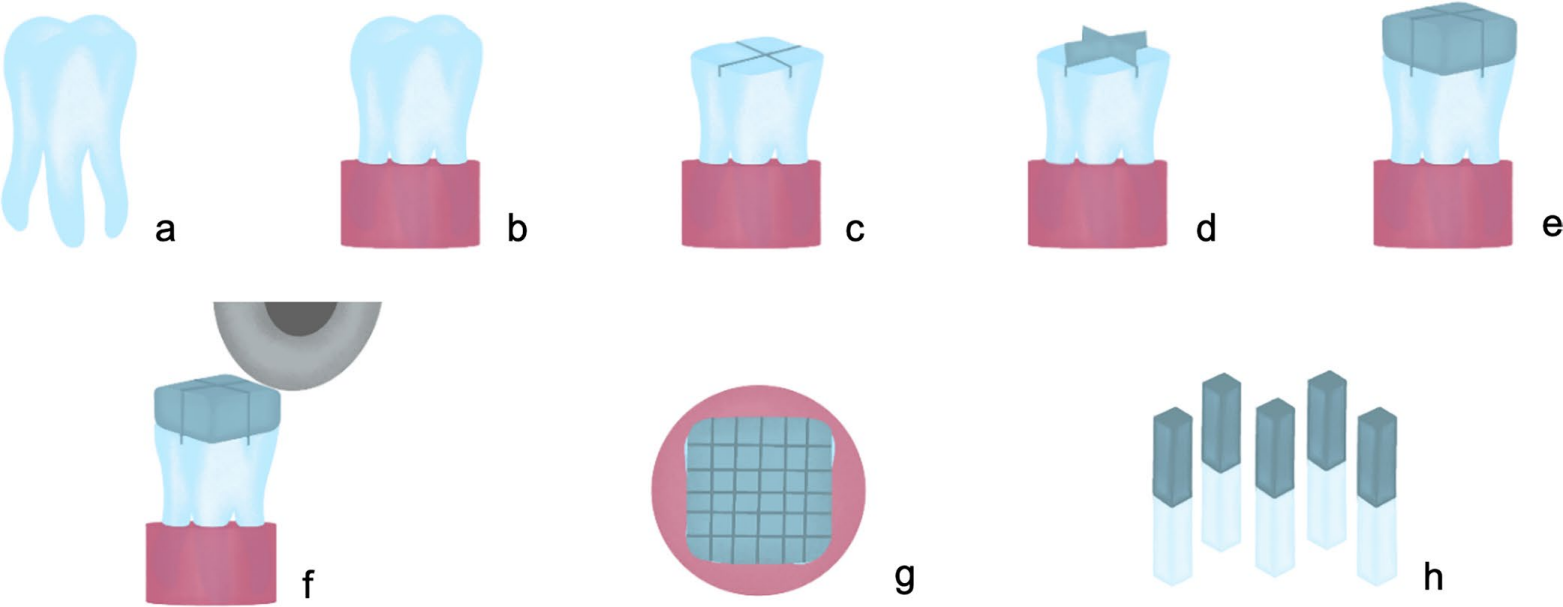
Schematic representation of the successive steps for tooth preparation, restorative procedure and preparation for microtensile bond strength testing: (a) initial tooth; (b) after inclusion in auto‐polymerizing acrylic resin; (c) divided sample; (d) metal matrices in place; (e) restorative procedures completed; (f) sample sectioning; (g) occlusal view after sectioning; (h) resin‐dentin sticks.

Base prototypes were 3D printed in a hard PMMA‐like resin (Dental Pink, HARZ Labs LLC., Moscow, Russia) using PrusaSlicer software (Prusa Research, Prague, Czech Republic) to ensure predictability in tooth positioning and a 90° angle between surfaces in the cutting steps. Specimens were fixed to these bases by partial inclusion of the roots with auto‐polymerizing acrylic resin (Schmidt Laboratory, Madrid, Spain, Lot: 47975, Exp: 2024/11), ensuring occlusal surfaces remain parallel to the horizontal plane (Figure 1a,b).

Using an Accutom 5 machine (Accutom 5, Struers, Ballerup, Denmark) with an integrated cooling system, a crown section perpendicular to the long axis of the tooth was performed at the coronal equator to expose a flat dentinal surface. Under ×40 magnification (Leica M300, Leica Microsystems, Heerbrugg, Switzerland) peripheral enamel was removed with a diamond bur in an air turbine handpiece. Afterwards, dentinal surfaces were manually prepared with a #600‐grit silicon carbide (SiC) abrasive paper in a circular motion for 30 s, under constant irrigation, to create a standardized smear layer [36]. All experimental procedures were performed by one experienced operator.

The specimens were randomly divided into two groups (*n* = 10 for each group) [36], according to the adhesive strategy: etch&rinse and self‐etch. Each tooth was further divided into four equal parts, corresponding to four subgroups, submitted to different dentin surface treatments. This division eliminated testing upon different substrates and was achieved with two perpendicular thin and shallow grooves, using the previously mentioned sectioning machine (Accutom 5, Struers, Ballerup, Denmark) (Figure 1c). Metal matrices (Hawe, Kerr, Orange, CA, USA), 6‐mm high, were placed inside the grooves and stabilized with flowable resin to individualize each quarter of the tooth for the airborne‐particle abrasion procedures (Figure 1d). The eight experimental groups are described in Table 1.

**TABLE 1:** Experimental groups.

Group	Bonding system	Dentin pretreatment
OFL/Control	OptiBond FL	No pretreatment
OFL/Al_2_O_3_/Aquasol		Airborne‐particle abrasion with aluminum oxide, with irrigation
OFL/Al_2_O_3_		Airborne‐particle abrasion with aluminum oxide, without irrigation
OFL/BG45S5		Airborne‐particle abrasion with bioactive glass 45S5
CSEB/Control	Clearfil SE Bond	No pretreatment
CSEB/Al_2_O_3_/Aquasol		Airborne‐particle abrasion with aluminum oxide, with irrigation
CSEB/Al_2_O_3_		Airborne‐particle abrasion with aluminum oxide, without irrigation
CSEB/BG45S5		Airborne‐particle abrasion with bioactive glass 45S5

Airborne‐particle abrasion procedures were performed with Aquacare (Velopex International, London, UK) at a pressure of 4 bar, for 5 s. The device's tip was positioned 5 mm from the surface, with a 45° inclination. In groups OFL/Al_2_O_3_/Aquasol, OFL/BG45S5, CSEB/Al_2_O_3_/Aquasol, and CSEB/BG45S5, airborne‐particle abrasion was carried out under simultaneous continuous irrigation with AquaSol (AquaSol, Velopex International, London, UK), a water‐based solution containing 17.5% ethanol.

In addition to metal matrices, teflon was used to shield the untargeted dentin surfaces, thus preventing cross‐contamination among subgroups. After airborne‐particle abrasion procedures, specimens were copiously washed for 60 s with a stream of distilled water and air to remove the remaining abrasive powder from the surface, and then dried.

### 2.2 Bonding and Restorative Procedures

One blind‐to‐the‐groups and experienced operator performed all the following described procedures. The two adhesive systems used, Clearfil SE Bond (CSEB; self‐etch approach; Kuraray Noritake Dental Inc., Okayama, Japan) and OptiBond FL (OFL; etch&rinse approach; Kerr Corporation, CA, USA) were applied according to the manufacturer's recommendations, simultaneously to all groups within the same tooth. Materials, manufacturers, chemical composition, application procedure, and lot numbers are described in Table 2. Afterward, a flowable composite (GrandioSO Heavy Flow, Voco GmbH, Cuxhaven, Germany, Lot: 2218399, Exp: 2024/08) layer was applied and light‐cured, followed by the build‐up with a composite resin (Ceram.x Spectra ST, Dentsply Sirona, Konstanz, Germany, Lot: 2208000500, Exp: 2025/07), in three increments with approximately 1.5 mm (Figure 1e), each light‐cured for 30 s (Bluephase Style 20i, Ivoclar Vivadent, Schaan, Liechtenstein, 1200 mW/cm^2^). After bonding procedures completion, matrix bands were removed, and the teeth were stored in distilled water at 37°C for 1 month.

**TABLE 2:** Materials, manufacturers, chemical composition, application procedure, and lot numbers.

Material	Manufacturer	Composition	Application procedure	Lot number
Clearfil SE Bond	Kuraray Noritake Dental Inc., Kurashiki, Okayama, Japan	Primer: 10‐MDP, HEMA, hydrophilic aliphatic dimethacrylate, dl‐Camphorquinone, water Bond: 10‐MDP, Bis‐GMA, HEMA, hydrophobic aliphatic dimethacrylate, dl‐Camphorquinone, initiators, accelerators, silanated colloidal silica	Primer: active application for 20 s, followed by drying Bond: active application for 20 s, followed by drying Light‐cure for 30 s	0180
OptiBond FL	Kerr Corporation, Orange, CA, USA	Primer: 2‐hydroxyethyl methacrylate, ethanol, 2‐[2(methacryloyloxy) ethoxycarbonyl] benzoic acid, glycerol phosphate dimethacrylate Bond: glass, oxide, chemicals, 2‐hydroxyethyl methacrylate, Ytterbium trifluoride, 3‐trimethoxysilylpropyl methacrylate, 2‐hydroxy‐1,3‐propanediyl bismethacrylate, alkali fluorosilicates (Na)	Acid etching with 35% phosphoric acid (Ultra‐Etch, Ultradent Products Inc. UT, EUA) for 15 s Primer: active application for 20 s, followed by drying Bond: active application for 20 s, followed by drying Light‐cure for 30 s	8537244
GrandioSO Heavy Flow	Voco GmbH, Cuxhaven, Germany	Barium aluminum borosilicate glass, silicon oxide, HEDMA, BisGMA, TEGDMA, BisEMA, fumed silica, initiators, stabilizers, pigments	Placement of a thin layer (~0.3 mm thick) of flowable composite and light‐cure it for 30 s	2218399
Ceram.x. Spectra ST Low Viscosity	Dentsply Sirona, Konstanz, Germany	Ethoxylated bisphenol A dimethacrylate, urethane modified Bis‐GMA dimethacrylate resin, 2,2‐ethylenedioxydiethyl dimetharcylate, ytterbium trifluoride, 2,6‐ditert‐butyl‐*p*‐cresol	Placement of several increments with approximately 1.5 mm, each one light‐cured for 30 s	2208000500
proCut (29 μm Al_2_O_3_)	Medivance Instruments Ltd., London, UK	Aluminum oxide	Airborne‐particle abrasion at 4 bar, for 5 s and tip‐to‐surface distance of 5 mm, with 45° inclination, with or without irrigation	200618
proSylc (Sylc bioglass 45S5)	Dentofex Research Ltd. London, UK	45S5 Bioglass: Silicon, calcium, sodium, phosphorus and oxygen	Airborne‐particle abrasion at 4 bar, for 5 s and tip‐to‐surface distance of 5 mm, with 45° inclination, with irrigation	021018

### 2.3 Microtensile Bond Strength

After the storage period, eight specimens of each group were cross‐sectioned (Accutom 5, Struers, Ballerup, Denmark) perpendicularly to the bonding interface, under water refrigeration, at 300 rpm, with a feed of 0.50 mm/s, to produce sticks with a sectional area of approximately 0.9 mm^2^ (Figure 1f). After sectioning in one direction, the existent space between the slices was filled with light‐bodied silicone Aquasil Ultra XLV (Dentsply Sirona, Konstanz, Germany) prior to the second cut (Figure 1g). Lastly, a cut was made parallel to the bonding interface to release the sticks (Figure 1h). All samples were analyzed under ×40 magnification (Leica M300, Leica Microsystems, Heerbrugg, Switzerland), with the purpose of discarding any that could present defects, such as voids or surface defects. A total of three sticks were discarded, with a mean of 44 ± 3 sticks obtained per group.

Specimens were then submitted to a microtensile bond strength test in a universal testing machine (Model AG‐I, Shimadzu Corporation, Kyoto, Japan), at a 0.5 mm/min crosshead speed until failure. The maximum load at failure was registered in Newton (N) by the software Trapezium (Shimadzu Corporation, Kyoto, Japan) and was then divided by the cross‐sectional area of each stick (mm^2^), measured with a digital caliper, to allow the calculation of the bond strength at failure (MPa).

### 2.4 Failure Mode Analysis

After testing, the mode of failure was analyzed by two experienced operators under ×40 magnification (Leica M300, Leica Microsystems, Heerbrugg, Switzerland) and classified as adhesive, cohesive in dentin, cohesive in composite or mixed. While adhesive failures occur at the bonding interface, cohesive failures are the ones within dentin or composite, without affecting the interface. Mixed failures are those involving both the bonding interface and the substrate/restorative material.

### 2.5 Scanning Electron Microscopy (SEM)

Prior to bonding and restorative procedures, two specimens of each group were fixed in 2.5% glutaraldehyde in a 0.1 M sodium cacodylate buffer solution (pH 7.4) for 12 h at 4°C, and rinsed with 0.2 M sodium cacodylate buffer solution (pH 7.4) for 1 h (with three changes). Subsequently, a dehydration process was conducted in an ascending ethanol series: 25%, 50%, and 75% for 20 min each; 95% for 30 min; and 100% for 60 min [37, 38]. Specimens were then immersed in hexamethyldisilane for 30 min, dried and stored in absorbent paper at room temperature for 24 h. After fracture to allow longitudinal and transversal observation, samples were sputter‐coated with gold (MED 010, Balzers Union, Balzers, Liechtenstein) and observed in a scanning electron microscope in secondary mode at 15 kV (SEM, VEGA 3 TESCAN, Shimadzu, Tokyo, Japan). Photomicrographs of representative surface areas were taken.

### 2.6 Data Analysis

The bond strength for each study group was described using the mean, standard deviation, median, and 25th and 75th percentiles. Statistical analysis of bond strength between each adhesive system and preparation strategy was conducted using a linear mixed regression model, motivated by the fact that the preparation strategies were tested on the same tooth, with multiple trials performed for each condition. Therefore, the adhesive system was considered a fixed effect, while the different pretreatments were treated as repeated measures within each tooth. The sticks were considered independent observations nested within each condition and the trials conducted under each condition were modeled as random effects.

Failure mode was examined independently for each adhesive system, comparing the different pretreatments and, for each pretreatment, a comparison between both adhesive systems was conducted. Each condition, considering the adhesive system and the preparation strategy, was defined by the absolute and relative frequency of each failure mode. Fisher's exact test was carried out to compare the adhesive systems within each preparation strategy. Different strategies, for each adhesive, were assessed by Cochran's *Q* test, considering adhesive failures in relation to other failure modes.

The statistical analysis was performed using IBM SPSS v28 (IBM Corp., NY, USA), with the significance level set at 0.05.

## 3 Results

### 3.1 Microtensile Bond Strength

**FIGURE 2:**
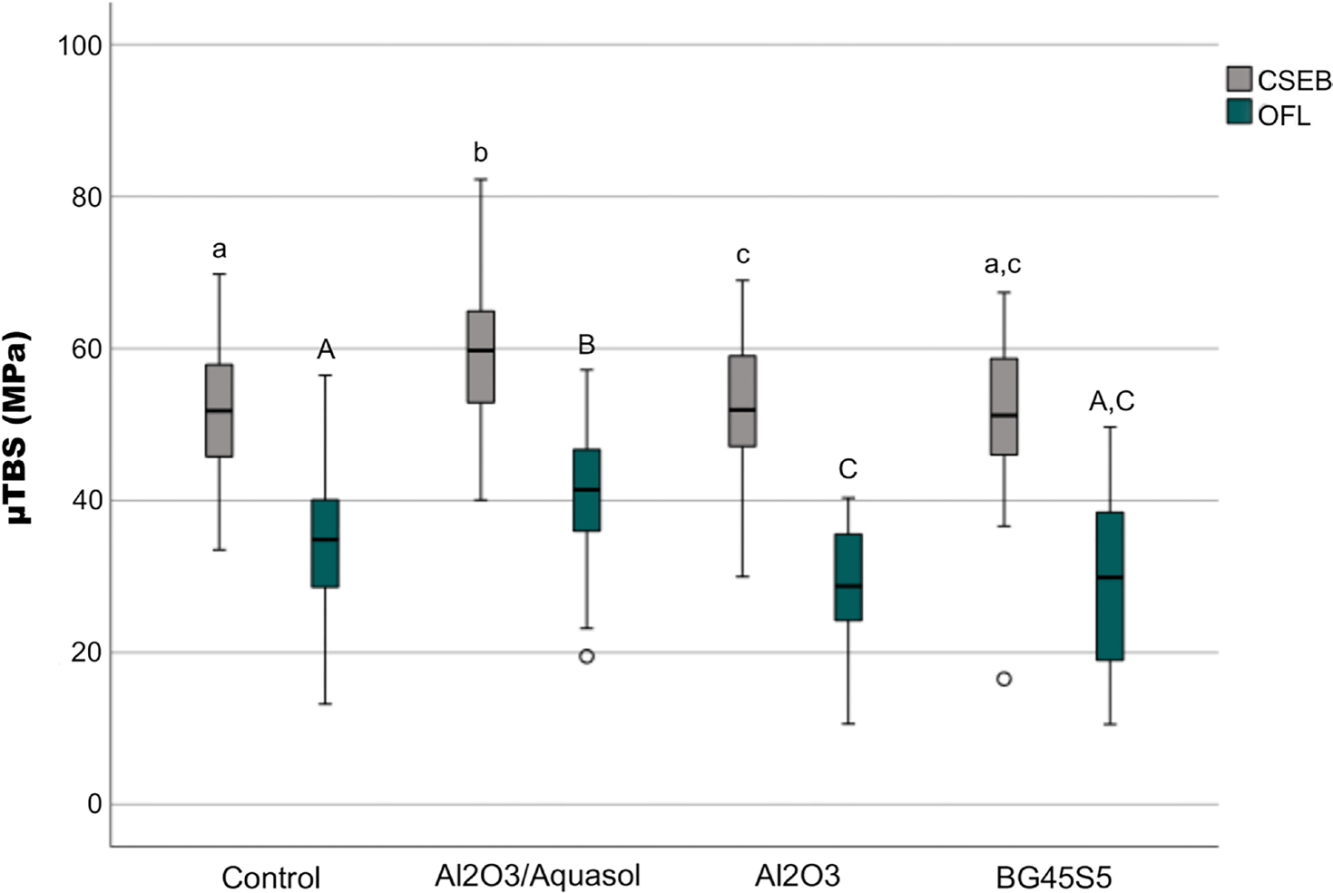
Box plots illustrate the distribution of microtensile bond strength (μTBS) values in the study groups. Different uppercase and lowercase letters indicate statistically significant differences between the study groups for Clearfil SE Bond (CSEB) and Optibond FL (OFL), respectively (*p* < 0.05).

Regarding μTBS, the linear mixed regression model reveals statistically significant differences concerning both the adhesive strategy (*p* < 0.001) and the applied pretreatment (*p* < 0.001). Airborne‐particle abrasion procedures involving Al_2_O_3_ (Al_2_O_3_/Aquasol and Al_2_O_3_) exhibit statistically significant differences compared to the control (*p* < 0.001 and *p* = 0.006, respectively) (Figure 2). Additionally, Al_2_O_3_/Aquasol resulted in statistically higher μTBS than the other tested pretreatments, Al_2_O_3_ and BG45S5 (*p* < 0.001), whereas BG45S5 rendered similar μTBS values when compared to the control (*p* = 0.080) and Al_2_O_3_ groups (*p* = 0.467).

Relative frequencies of the observed failure modes are shown in Table 3. No statistically significant association was observed between failure mode and the adhesive strategy in the control group (Fisher's exact test, *p* = 0.071) and in the BG45S5 group (Fisher's exact test, *p* = 0.118). Al_2_O_3_/Aquasol and Al_2_O_3_ groups exhibit a statistically significant association (Fisher's exact test, *p* = 0.002 and *p* = 0.005, respectively) between the applied adhesive and failure mode. Statistically significant differences were observed between adhesive failures and the different preparation strategies for CSEB (*p* < 0.001) and for OFL (*p* = 0.002).

**TABLE 3:** Microtensile bond strength (μTBS) values and relative frequencies of the observed failure modes for the two adhesive strategies with different pretreatments.

Adhesive system	Pretreatment	μTBS (MPa)^a^	Failure mode (%)		
			Adhesive	Cohesive in dentin	Cohesive in composite
Clearfil SE Bond	Control	51.7 (9.1) 45.8/57.9	82.2	11.1	6.7
	Al_2_O_3_/Aquasol	59.1 (8.8) 52.9/64.9	32.7	26.5	40.8
	Al_2_O_3_	52.2 (10.0) 46.5/59.1	81.4	9.3	9.3
	BG45S5	51.3 (9.8) 45.9/59.6	90.7	0	9.3
Optibond FL	Control	33.9 (8.9) 28.6/40.1	93.5	0	6.5
	Al_2_O_3_/Aquasol	41.0 (8.6) 36.0/46.7	68.9	11.1	20.0
	Al_2_O_3_	28.5 (8.1) 24.2/36.9	100	0	0
	BG45S5	30.1 (11.2) 17.6/40.0	100	0	0

^a^ μTBS: mean (standard deviation) 25th percentile/75th percentile.

### 3.2 SEM Observations

**FIGURE 3:**
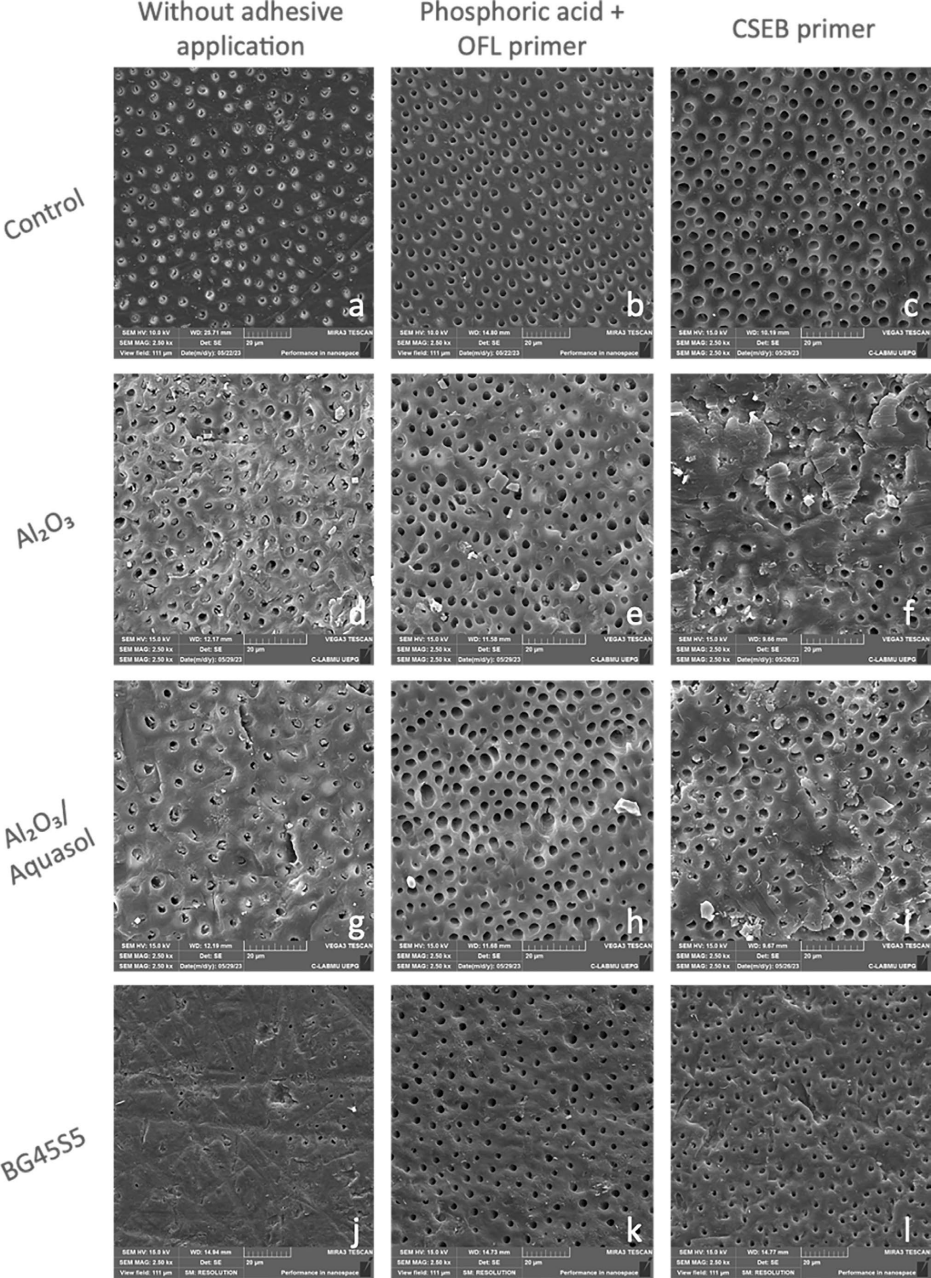
Representative SEM images of dentin surfaces at ×2500 magnification. (a) untreated dentin; (b) OFL/Control; (c) CSEB/Control; (d) Al_2_O_3_; (e) OFL/Al_2_O_3_; (f) CSEB/Al_2_O_3_; (g) Al_2_O_3_/Aquasol; (h) OFL/Al_2_O_3_/Aquasol; (i) CSEB/Al_2_O_3_/Aquasol; (j) BG45S5; (k) OFL/BG45S5; (l) CSEB/BG45S5.

Untreated dentin (Figure 3a) exhibits an intertubular smear layer, partially obstructing the majority of dentinal tubules. After the adhesive system application (Figure 3b,c), both the smear layer and smear plugs were removed, resulting in widened dentin tubules.

Dentin airborne‐particle abrasion with Al_2_O_3_ without irrigation (Figure 3d) produces a dense, compact and amorphous smear layer, as well as a cratered surface with most tubules obstructed. Furthermore, crack‐like alterations can be observed along the surface. In OFL/Al_2_O_3_ samples (Figure 3e), after acid etching and primer application, the smear layer is mostly removed, but some debris, residual particles, and densification of the dentin surface remain visible. CSEB/Al_2_O_3_/Aquasol samples (Figure 3f) show both open and obliterated tubules, with debris inside and occasional abrasive particles.

Al_2_O_3_ with Aquasol airborne‐particle abrasion (Figure 3g) yields an irregular smear layer partially occluding the tubule entrances, but with fewer debris compared to the non‐irrigated procedure. OFL/Al_2_O_3_/Aquasol samples (Figure 3h) present widened tubules without smear layer, no signs of surface compaction and few residual particles. CSEB/Al_2_O_3_/Aquasol images (Figure 3i) reveal the existence of some airborne‐particle abrasion debris and abrasive particles on the surface.

BG45S5 airborne‐particle abrasion (Figure 3j) results in a dense smear layer partially obstructing the tubules. After acid etching and primer application, OFL/BG45S5 samples (Figure 3k) display diminished deposition of the smear layer, although its compaction remains evident. CSEB/BG45S5 samples (Figure 3l) exhibit the presence of some debris and also a dense smear layer with some obliterated tubules. Retained abrasive clusters in dentin are fewer and smaller than those in groups using Al_2_O_3_.

## 4 Discussion

### 4.1 Dentin Adhesion

Both research hypotheses were accepted as some of the tested airborne‐particle abrasion techniques significantly affected dentin bond strength and all of them resulted in distinct findings under scanning electron microscopy.

Bonding to dentin relies not only on the mineral content of intertubular dentin and proximity to the pulp, but also on the produced smear layer thickness and density, due to the major influence on adhesive system penetration [11, 18, 20]. In this study, #600‐grit SiC paper was used with the purpose of creating a uniform and standard smear layer [36]. As opposed to bur‐prepared surfaces, this procedure provides a layer with fewer flaws and irregularities [15, 39], as well as a higher number of open dentinal tubules [39].

Both adhesive systems used in the present study, OptiBond FL [1, 2] and Clearfil SE Bond [2], are considered the “gold standard” regarding etch&rinse and self‐etch adhesives, respectively, as they are associated with higher effectiveness and bonding durability. In SEM analysis of OFL/Control and CSEB/Control, the standard smear layer created upon untreated dentin was successfully removed or dissolved, respectively, and dentin tubules were exposed, allowing subsequent bonding interactions with the bonding resin.

In what concerns bonding performance, Clearfil SE Bond presented higher bond strength values than OptiBond FL (*p* < 0.001), as the self‐etch system presents less sensitivity and enhanced predictability. Consistent with these results, de Munck et al. reported higher dentin bond strength values for Clearfil SE Bond in comparison with OptiBond FL, regardless of the storage period (1 week, 3 months, 6 months, and 12 months) [40].

Phosphoric acid application, required by the etch&rinse technique, induces alterations in dentin morphology and permeability [41, 42], with a major disadvantage based on the lack of standardization in substrate moisture after rinsing and drying steps, as etched dentin desiccation causes the collagen network to collapse and excessive humidity hinders adhesive monomers' penetration, inducing the formation of voids at the bonding interface and decreasing the degree of resin monomer conversion [2, 7, 19, 43]. Moreover, collagen can remain unencapsulated by the adhesive, increasing the susceptibility to degradation and jeopardizing the long‐term hybrid layer establishment [2, 6, 15, 34, 44]. Furthermore, phosphoric acid leads to matrix metalloproteinases' activation, also contributing to the degradation of the uninfiltrated collagen layer through collagenolytic and gelatinolytic activity [5, 32, 39].

Contrariwise, the bonding performance of self‐etch adhesives is dependent on the maintenance of the adhesive acidic potential after contact with the mineral content of the smear layer, as there is a tendency for buffering action and, consequently, an increase in pH value [2, 6, 45, 46]. Mild self‐etch adhesives with 10‐MDP functional monomers, such as Clearfil SE Bond, have the advantage of chemical interaction with calcium present in hydroxyapatite, which alongside the achieved micromechanical retention, provides an increase in bond strength and higher predictability, as well as higher resistance to debonding stress and hydrolysis [1, 2, 5, 15].

Among all experimental groups, statistically significant differences between adhesive failures and the different preparation strategies were observed for Clearfil SE Bond (*p* < 0.001) and OptiBond FL (*p* = 0.002). Whereas adhesive failures were the predominant type, causing disruption of the adhesive interface, for Clearfil SE Bond, adhesive failures were replaced as the most prevalent, in one of the groups, by cohesive failures. Cohesive fractures are associated with higher bonding performance as the cohesive strength of the materials (dentin and composite resin) fails before the adhesive interface, which is consistent with the obtained results.

### 4.2 Airborne‐Particle Abrasion With Aluminum Oxide

Bonding interactions are directly related to the smear layer obtained when preparing the substrate, which led to the development of different methods to achieve a smear layer with optimized features [8, 15, 26, 41]. Airborne‐particle abrasion is a procedure associated with the creation of surface irregularities to improve the contact between dentin and adhesive, thus providing additional mechanical retention [9, 13, 19, 47]. It is worth mentioning that airborne‐particle abrasion procedures have been shown to preserve dentinal tubules' original diameter and intertubular dentin [8, 47].

Al_2_O_3_ particles are considered the most abrasive, as they are irregularly shaped and have higher hardness values [34]. Whereas alumina has, approximately, a Young's modulus of 380 GPa and Vickers hardness of 2300 VHN, bioactive glass is characterized by lower values, of 35 GPa and 458 VHN, respectively. The dentinal substrate is known to have even lower Young's modulus and Vickers hardness values [21, 33, 48].

SEM images of air‐abraded dentin are characterized by the presence of an irregular, dense and amorphous smear layer, occluding the majority of tubules, as previously observed by other authors [13, 49]. Furthermore, crack‐like alterations can be observed along the surface. In the OFL/Al_2_O_3_ samples, phosphoric acid application was able to generally remove this layer, but a compact dentinal surface is present even after primer application, alongside some particle remnants. On the other hand, in the CSEB/Al_2_O_3_ samples, both open and obstructed tubules can be observed, as well as debris and residual abrasive particles on the dentin surface.

According to microtensile results, the Al_2_O_3_ samples exhibit statistically significant differences (*p* = 0.006) compared to the control. Despite the significant difference in both OFL/Al_2_O_3_ and CSEB/Al_2_O_3_ samples, CSEB is associated with higher bond strength values and OFL, on the other hand, resulted in lower bonding performance than the respective control.

The present results, regarding the use of an etch&rinse strategy, are in accordance with Pahlavan et al. who reported a decrease in bonding performance, associated with the detrimental effect of the remaining abrasive particles [13]. Even though airborne‐particle abrasion procedures increased the area for bonding interactions [4, 8, 47], it has been stated that surface roughness does not significantly affect bond strength values [7]. Additionally, Coli et al. concluded that, besides roughness, other parameters must be considered, such as the chemical composition of the substrate [9]. Regarding the remaining residual Al_2_O_3_ particles, Sutil and Susin demonstrated that these clusters can hinder the contact between dentin and the adhesive, harming bond strength values [19].

Regarding the use of a self‐etch strategy, a major factor to consider is the acidic primer's ability to dissolve and incorporate a much denser and irregular smear layer. Available literature is nonconsensual about the effect of Al_2_O_3_ airborne‐particle abrasion on bonding performance when using self‐etch adhesives, with the majority of the studies reporting that this procedure does not enhance or impair bond strength values [28].

The failure mode observed in our experimental groups was mostly adhesive, with a statistically significant association between the applied adhesive and failure mode (*p* = 0.005). The CSEB/Al_2_O_3_ group exhibited 81.4% adhesive and 18.6% cohesive failures, in contrast to the OFL/Al_2_O_3_ group, which had 100% adhesive failures. Consistent with the results of this study, Burnett Jr. et al. evaluated the tensile bond strength of a universal adhesive system placed over differently pretreated dentin to conclude that the Al_2_O_3_ airborne‐particle abrasion group had a predominance of adhesive failures with, approximately, 73%, followed by 27% of cohesive failures [50].

As previously stated, over the last few years there has been a discernible proclivity toward the inclusion of concomitant irrigation during the conveyance of abrasive particles, with the advantage of regulating their dispersion [27]. Regarding Al_2_O_3_ airborne‐particle abrasion with simultaneous irrigation, SEM images reveal, in the OFL/Al_2_O_3_/Aquasol group, the total removal of the smear layer created by the previously applied pretreatment. Dentin tubules become wide open, with the presence of few residual abrasive particles. On the other hand, in the CSEB/Al_2_O_3_/Aquasol group, some particle residue and debris are seen on the surface.

It is worth mentioning that all SEM images of air‐abraded dentin with Al_2_O_3_ under continuous irrigation present a more regular smear layer and a reduced number of particles, when comparisons are made with the same procedure without irrigation. Paolinelis et al. studied the retention of bioactive glass on dentin to conclude that, when water is used during the airborne‐particle abrasion procedure, not only is dust reduced but also the amount of abrasive retained [22]. Although the abrasive powder is not the same, deductions that the fluid released simultaneously is the main responsible for this result can be made.

Microtensile bond strength results of both OFL/Al_2_O_3_/Aquasol and CSEB/Al_2_O_3_/Aquasol groups show statistically significant differences (*p* < 0.001) with the respective control, meaning that the airborne‐particle abrasion procedure accomplished under irrigation resulted in higher bond strength values for both adhesive strategies. Furthermore, these groups also obtained statistically significant higher bond strength values than the groups air abraded without irrigation (*p* < 0.001) and air abraded with bioactive glass (*p* < 0.001), for each adhesive system. These results can be explained by the production of a less compacted, debris‐packed smear layer which is then almost eliminated, and by the greater opening of dentinal tubules, allowing adhesive systems to operate effectively.

The present results are in accordance with Mavriqi et al. that evaluated the efficacy of water‐airborne‐particle abrasion, when bonding with an etch&rinse system, to conclude this procedure is associated with an increase of 23.6% in bond strength, in comparison to the control group, only treated with acid etching [27]. On the other hand, Spagnuolo et al. reported similar bond strength values between airborne‐particle abrasion with Al_2_O_3_, using Aquacare, and the control group (#320‐grit SiC paper), applying a universal adhesive in both self‐etch and etch&rinse mode [31].

Furthermore, the predominant failure mode was cohesive failures, which can be directly correlated to the fact that the bond strength of adhesive systems is stronger than the cohesive resistance of the materials (dentin and composite resin). For Al_2_O_3_/Aquasol a statistically significant association (*p* = 0.002) was found between failure mode and the adhesive applied, meaning that despite the satisfactory results obtained in both groups, Clearfil SE Bond was associated with better performance due to the higher predominance of this failure mode (67.3%).

### 4.3 Airborne‐Particle Abrasion With Bioactive Glass

Bioactive glasses have previously demonstrated their high biocompatibility and remineralization potential, involving different ions, such as sodium (Na^+^), calcium (Ca^2+^), and phosphate (PO_4_^3−^) [51]. Upon exposure to surrounding oral fluids, there is leaching of Na^+^, within the first minute, and dissolution of Ca^2+^, PO_4_^3−^, and Si^4+^ on the surface of the particles, leading to subsequent precipitation of an amorphous calcium phosphate layer, further converted into hydroxyapatite [Ca_10_(PO_4_)_6_(OH)_2_] [32, 51, 52, 53, 54]. Additionally to this process, ionic exchanges lead to an increase in pH and consequent disruption of bacterial cytoplasmic membranes and reduction of the proteolytic action of endogenous matrix metalloproteinases, explaining the antibacterial action of bioactive glasses [52, 55].

SEM observations show that using bioglass 45S5 for airborne‐particle abrasion resulted in a consistent compact smear layer across all samples, independently of the performed surface treatment, which partially occluded the dentinal tubules. In the OFL/BG45S5 samples, the smear layer was removed, with a lesser amount of abrasive retained. In the CSEB/BG45S5 samples, the system's primer dissolved part of the smear layer. Residual debris and abrasive particles can be found, compared to the Al_2_O_3_ samples. Consistent with the results of this study, Paolinelis et al. concluded that, for the same abrasion parameters, alumina was more retained on dentin in comparison with bioactive glass, as the latter has a higher tendency for fracture due to its brittleness [22]. It is worth mentioning that bioactive glass, when retained, has the apparent advantage of remineralization and antibacterial properties [22].

According to our results, the BG45S5 group presents bond strength values with no statistically significant difference compared to the control (*p* = 0.080). Thus, divergences in SEM images did not translate into differences regarding microtensile results. Furthermore, no statistically significant differences were found between BG45S5 and Al_2_O_3_ (*p* = 0.467). Additionally, the BG45S5 group presented no statistically significant association (*p* = 0.118) between failure mode and adhesive strategy, as both groups present a prevalence of adhesive failures higher than 90%.

Spagnuolo et al. demonstrated that there is no negative effect on the bonding performance when etch&rinse system is used, despite the higher resistance to acid etching reported [31]. Furthermore, it was observed that specimens subjected to airborne‐particle abrasion with BG45S5 in the self‐etch experimental group exhibited decreased bond strength values. This can be attributed to the alkaline nature of bioglass, which can persist within the smear layer and impair the bonding efficacy of adhesives employed in self‐etch mode, since acidic functional monomers lack comparable effectiveness in such circumstances [31].

On the other hand, Sauro et al. reported that airborne‐particle abrasion with bioactive glass can result in increased bond strength when adhesive systems containing 10‐MDP are used, due to the greater number of ionic bonds between adhesive monomers and calcium [48].

### 4.4 Considerations and Limitations

When operating with abrasive powders, it is imperative to consider the necessity of minimizing occupational hazards. This can be achieved not only through rubber dam isolation, but also by employing eye protective gear and an appropriate evacuation system [56]. These precautionary measures are necessary to prevent the spread of particles and should be complemented by irrigation during airborne‐particle abrasion procedures.

Airborne‐particle abrasion pretreatment influences dentin morphology, the type of smear layer created and, consequently, bonding interaction between the adhesive and dentinal substrate. Available literature on the use of airborne‐particle abrasion as a prebonding dentin surface treatment remains insufficient to achieve solid conclusions, mostly due to the existing contradictory results.

Moving forward, it is imperative that research delves further into the topic with aged specimens to comprehensively evaluate the impact of pretreatment on long‐term bonding success and stability to dentin. Additionally, since sound dentinal substrates are scarce in daily clinical practice, this procedure should be tested upon altered substrates, such as sclerotic, demineralized, and eroded dentin.

## 5 Conclusion

Different abrasive powders render distinct bond strength results to dentin substrate and reveal varying surface characteristics under scanning electron microscopy:

- The use of Al_2_O_3_ with simultaneous irrigation resulted in the highest bond strength values. On the other hand, bond strength outcomes of airborne‐particle abrasion with Al_2_O_3_ without irrigation directly depend on the used adhesive strategy, with the etch&rinse approach yielding the lowest results. Bioactive glass did not interfere with bonding performance of either tested adhesive systems.
- The use of continuous irrigation reduced debris accumulation and enhanced tubule exposure in the groups subjected to airborne‐particle abrasion with Al_2_O_3_. Bioactive glass produced a compact smear layer with fewer retained abrasive residues than the Al_2_O_3_ groups.

## Funding

The authors have nothing to report.

## Disclosure

The authors have nothing to report.

## Conflicts of Interest

The authors declare no conflicts of interest.

## Data Availability

The data that support the findings of this study are available from the corresponding author upon reasonable request.
